# Listing all sorting reversals in quadratic time

**DOI:** 10.1186/1748-7188-6-11

**Published:** 2011-04-19

**Authors:** Krister M Swenson, Ghada Badr, David Sankoff

**Affiliations:** 1 Department of Mathematics and Statistics, University of Ottawa, Ontario, K1N 6N5, Canada; 2 LaCIM, UQAM, Montréal Québec, H3C 3P8, Canada; 3 SITE, School of Information Technology and Engineering, University of Ottawa, Ontario, K1N 6N5, Canada; 4 IRI - Mubarak city for Science and Technology, University and Research District, P.O. 21934 New Borg Alarab, Alex, Egypt

## Abstract

We describe an average-case *O*(*n*^2^) algorithm to list all reversals on a signed permutation *π *that, when applied to *π*, produce a permutation that is closer to the identity. This algorithm is optimal in the sense that, the time it takes to write the list is Ω(*n*^2^) in the worst case.

## 1 Introduction

In 1995 Hannenhalli and Pevzner [1] presented an algorithm to transform one genome into another in a minimum number of biologically plausible moves. They modeled a genome as a signed permutation and the move that they considered was the reversal: the order of a substring of the permutation is reversed, and the sign of each element in the substring is flipped. Since then many refinements and speed improvements have been developed [2-8].

In 2002 Siepel and Ajana et al. [9,10] showed how to list every parsimonious scenario of reversals, each scenario being a proposed candidate for the true evolutionary history. Fundamental to their algorithms are *O*(*n*^3^) techniques for finding all *sorting *reversals; the reversals that at each step produce a permutation that is closer to the target permutation than the last. Ajana et al. [9] used these results to support the replication-directed reversal hypothesis. Lefebvre et al. [11] and Sankoff et al. [12] used similar methodology to gain insight into the distribution of reversal lengths between genomes. Algorithms that attempt to more succinctly represent all shortest-length scenarios [13,14] have also been developed.

In this paper we show how to list all sorting reversals in *O*(*n*^2^) time on average. This algorithm is optimal in the sense that there are Ω(*n*^2^) safe cycle-splitting reversals in the worst case. We later give a family of permutations that have Ω(*n*^2^) unsafe reversals.

We implemented our algorithm in Java, and show experimentally that our algorithm is significantly faster than that of Siepel. This will afford a marked speedup of the aforementioned methods [9-14], since listing all sorting reversals is the kernel of repeated computation in each of them, especially when applied to permutations of sizes 3 × 10^3 ^to 3 × 10^5 ^(the size of bacterial or mammalian genomes).

After giving background material in Section 2 we introduce ominous substrings in Section 3. Section 4 describes how to detect the set of all ominous substrings of a permutation efficiently while Section 5 presents the algorithm. Section 6 shows the empirical speedup that our implementation affords. Finally, Section 7 gives a family of permutations that have Ω(*n*^2^) unsafe reversals and discusses open problems.

## 2 Background

Take a signed permutation *π *= *π*_1_,..., *π_n _*on the integers from 1 to *n*. Define a (signed) *reversal ρ*(*i, j*) as the signed permutation

That way, applying the reversal *ρ*(*i, j*) to permutation *π *gives

Given signed permutations *π*_1 _and *π*_2_, the *reversal distance d*(*π*_1_, *π*_2_) is the smallest *k *such that *π*_2 _= *π*_1 _○ *ρ*_1 _○ *ρ*_2 _○ ··· ○ *ρ_k_*. Since , we consider *π*_2 _= *I *= 1, 2,..., *n *to be the identity permutation.

In this paper, we describe our methods using circular permutations (when written on a line, the leftmost element follows the rightmost element), as any sorting reversal on a circular permutation has its counterpart on a linear version of the permutation. Occasionally, however, we refer to the *linearization *of a permutation *π*; this is a linear version of *π *that maintains the same ordering as the clockwise ordering of *π *but has a leftmost and a rightmost element.

### 2.1 All Sorting Reversals

A reversal *ρ *is a *sorting *reversal on *π *if *d*(*π *○ *ρ*) = *d*(*π*) - 1. Although the definition is simple, a characterization of all sorting reversals requires effort; to do so we must introduce the *breakpoint graph *[1]. Each element *π_i _*of permutation *π *has two vertices associated with it denoted by  and  (*π *^± ^can denote either). Embed the graph on a circle as follows: place all 2*n *vertices on the circle so that:

1.  and  are adjacent on the circle,

2.  is before (in the clockwise direction)  if and only if *π_i _*is positive, and

3. a  is adjacent to a  if and only if *π_i _*and *π*_*i*+1 _are adjacent in *π*.

**Figure 1:**
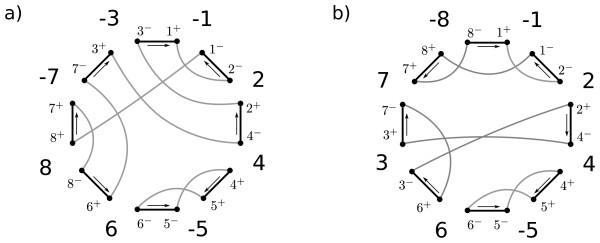
**Two breakpoint graphs**. The breakpoint graphs for a) *π *= (-1 2 4-5 6 8-7-3) and b) *π *○ *ρ*(6, 8). The direction that reality edges are traversed on a tour of the cycles is labeled with arrows. *ρ*(6, 8) is an unsafe reversal on *π*.

For two vertices  and  that are adjacent on the circle, add the edge (*v*_1_, *v*_2_)--a *reality *edge (also called a black edge); also add edges  for all *i *and --the *desire *edges (also called gray edges). Figure 1(a) shows the breakpoint graph for *π *= (-1 2 4-5 6 8-7-3). Note that every vertex has indegree 2 and outdegree 2, so the graph has a unique decomposition into cycles of even length (alternating between reality and desire edges).

A reversal *ρ*(*i, j*) is said to *act on *the reality edges  and  because these are the only edges in the breakpoint graph of *π *that are not in the graph of *π *○ *ρ*(*i, j*). In Figure 1, the reversal *ρ*(6, 8) acts on reality edges (3^-^, 1^+^) and (6^+^, 8^-^). Two reality edges on the same cycle are *convergent *if a traversal of their cycle visits each edge in the same direction in the circular embedding; otherwise they are *divergent*. The following definitions classify the action of a reversal on the cycles of the breakpoint graph [1].

**Definition 1 (cycle-splitting reversal) ***A reversal that acts on a pair of divergent reality edges splits the cycle to which the edges belong, so are called *cycle-splitting *reversals*.

Conversely, no reversal that acts on a pair of convergent reality edges splits their common cycle. A reversal that acts upon a pair of reality edges in two different cycles merges the two cycles. The permutation of Figure 1(a) has 10 cycle-splitting inversions including *ρ*(1, 2), *ρ*(4, 4), and *ρ*(6, 8). Notice that at most one cycle can be created by a reversal, yielding the inequality(1)

where *c*(*π*) is the number of cycles in the breakpoint graph. Most cycle-splitting reversals are sorting reversals [15], but not all sorting reversals are cycle-splitting reversals, which indicates a gap between this lower bound and the reversal distance.

We must further explore structure in the permutation that allows us to predict the reversal distance when the lower bound is not realized.

**Definition 2 (FCI **[16]) *A *framed common interval *(FCI) of a permutation (made circular by considering the first and last elements as being adjacent) is a substring of the permutation, as*_1_*s*_2 _... *s_k_b or *-*bs*_1_*s*_2 _... *s_k_*-*a such that*

• *for each i*, 1 ≤ *i *≤ *k*, |*a*| < |*s_i_*| < |*b*|, *and*

• *for each l*, |*a*| <*l *< |*b*|, *there exists a j with *|*s_j_*| = *l, and*

• *it is not a concatenation of substrings satisfying the previous two properties*.

So the substring *s*_1_*s*_2 _... *s_k _*is a (possibly empty) signed permutation of the integers that are greater than *a *and less than *b*; *a *and *b *are the *frame *elements, while those of *s*_1 _... *s_k _*are *trunk *elements if they are not trunk elements of a smaller FCI. The framed interval is said to be common, in that it also exists as an interval (*a*(*a *+ 1)(*a *+ 2)... *b*) in the identity permutation.

A *component *of a permutation is comprised of the trunk elements of an FCI that are not trunk elements of a shorter FCI, plus the frame elements. The permutation of Figure 1(b) has three components: one framed by elements 2 and 7, another framed by 4 and 6. The third is an interval in the circular sense, framed by elements 7 and 2 with the trunk comprised of elements 8 and 1; in the circular sense we have 7 < 8 < 1 < 2 here.

**Definition 3 (bad component **[16]) *A *bad component *of a permutation is a component with at least 4 elements, where the sign of every element is the same*.

In Figure 1(b), the component (2 4 6 3 7) is bad. The existence of one or more bad components in a permutation indicate exactly those situations where the lower bound cannot be met [1]. Siepel's paper [10] describes in detail an *O*(*n*^3^) algorithm for finding the set of sorting reversals when bad components exist. While further exploration of Siepel's characterization of sorting reversals in the presence of bad components could eventually lead to a worst-case *O*(*n*^2^) algorithm, we do not address the issue here. Suffice it to say that the average-case complexity is *O*(*n*^2^) even when the trivial *O*(*n*^3^) algorithm -- which in turn applies each of the *O*(*n*^2^) reversals and checks in linear time [17] if the distance has decreased -- is used on permutations with bad components. The probability that a permutation chosen uniformly at random has a bad component is *O*(*n*^-2^) [15,18] and we can detect the presence of bad components in linear time [16,17].

We focus on the bottleneck of sorting FCIs that do not correspond to bad components: cycle-splitting reversals that create bad components (cycle-splitting reversals that are not sorting reversals).

**Definition 4 (bad reversal) ***A *bad *reversal is a reversal that creates a bad component*.

**Definition 5 (unsafe reversal **[1]) *An *unsafe *reversal is a cycle-splitting reversal that is bad*.

In Figure 1(a), the reversal *ρ*(6, 8) is unsafe.

### 2.2 Outline

Known algorithms that list all sorting reversals check, one by one, if each of the potentially Ω(*n*^2^) cycle-splitting reversals is unsafe by applying the reversal and then running a linear time check as to whether it produced a bad (unoriented) component [9,10]. Instead of listing all cycle-splitting reversals and then checking them, we do the inverse: we predict which reversals may be *unsafe *(whether cycle-splitting or otherwise) and avoid listing them. We first characterize what we call *ominous *substrings of the permutation, those substrings that could be turned into a bad component with one reversal. Our algorithm searches for ominous substrings by doing the following: for each element of the permutation we posit that it is a smallest element of a potential (after a reversal) bad component and continue by scanning the permutation to detect an ominous substring.

## 3 Ominous Substrings

Take any unsafe cycle-splitting reversal *ρ *on permutation *π*. Since it is unsafe, the permutation *π *○ *ρ *has at least one bad component created by *ρ*. In this section we will show that there exists in *π *a particular pattern -- an *ominous *substring of *π *-- indicating that *ρ *is unsafe. We first describe ominous substring of permutations with a single component.

### 3.1 Permutations with a Single Component

A substring of a permutation is *ominous *if and only if there exists some elements *e *and *f *such that the substring fits one of the following templates (or their reverse):

1. (*eAX-f-B*): where *A*, -*B*, and *X *are substrings of the permutation. *A *has only positive while -*B *has only negative elements.

2. (*eA-BCf*): where *A*, -*B*, and *C *are substrings of the permutation. *A *and *C *have only positive while -*B *has only negative elements.

and *A *and *B *(and *C *if it exists) are comprised of exactly those elements with absolute value *i *for *e *<*i *<*f*.

In template 2, there already exists an FCI with frame elements *e *and *f*; the reversal that acts on exactly the elements of *B *fixes the elements of the interval to have the same sign. In the other template, a new interval is created with *e *and *f *as the frame elements, and {*f*} ∪ *B *∪ *X *are the elements reversed. For example, (-7 1-3-4-5-2 6) matches template 2 with the unsafe reversal acting upon the elements {2,3,4,5}; *A *and *C *are empty in this case. (-1 2 4 6-5-3) matches template 1 with the unsafe reversal acting upon the elements{3, 5, 6}; *f *= -5, -*B *= {-3}, and *X *= {6} in this case. (-2-6-8-4 1 5 7 9-3) matches the reverse of template 1 (*B f X*-*A*-*e*) with the unsafe reversal acting upon the elements {2, 3, 5, 7, 9} (or equivalently on the circular permutation, {1, 4, 6, 8}); -*A *= {-6, -8}, *B *= {5, 7}, and *X *= {-2, -3} in this case.

**Lemma 1 ***There is a one to one correspondence between bad reversals and ominous substrings*.

**Proof **By definition, there exists at least one reversal that creates a bad component from an ominous substring. On the other hand, take a permutation *π *○ *ρ *that has a bad component -- with frame elements *e *and *f *-- created by the reversal *ρ*. Say that the elements of the bad component are positive, then *e *is on the left and *f *is on the right. If *ρ *includes both *e *and *f*, this implies that the bad component already exists in *π*, which is a contradiction. Now let us examine the other three possibilities. If *ρ *does not include *e *and *f*, then the ominous substring in *π *corresponds to template 2. If *ρ *includes only *f*, then the ominous substring in *π *corresponds to template 1. If *ρ *includes only *e*, then the ominous substring in *π *corresponds to the reverse of template 1 where *ρ *acts upon the substring *XBf *(or equivalently, -*A *-*eY*, *Y *being the substring of *π *not matched by the reverse of template 1). If the elements of the bad component are negative then the negative analogue holds for each case. Since each ominous substring implies exactly one reversal dictated by the *A*, *B*, *C*, and *X*, we have the bijection.

### 3.2 Permutations with Multiple Components

We described ominous substrings on permutations with a single component. Since sorting reversals act only upon adjacencies in a single component [1], we adapt the techniques for single components to the case of multiple components in the following manner.

Consider a component of a permutation with some frame elements of a smaller FCI contained in it. We obtain the *condensed *version of the component by doing the following: for each smaller FCI contained in it, with pair of frame elements *a *and *b *(or -*a *and -*b*), we replace the FCI by *a *(resp. -*a*) and change the magnitude *m *of every element *m *>*b *in the component to be *m *- (*b*- *a*). The templates can be applied directly to the condensed component. For example, take the component *C *= (2 4 6 3 7) in Figure 1(b) where the component (4 -5 6) is contained in it. The condensed version of *C *is (2 4 3 5). The condensed version of any component can be computed in linear time.

## 4 Detecting Ominous Substrings

We now turn to the task of detecting an ominous substring associated with a smallest element *e*. The following methods can be adapted to detect the negative analogue of each template, so we only describe the detection of the templates as shown in Section 3.1. The general outline used in each of the following algorithms is the same: we visit the permutation starting with element *e*, proceeding to element *e *+ 1, then *e *+ 2 and so on. At each step we maintain enough information to check whether conditions that indicate we have found an ominous substring hold.

Call the set of elements that we visit through the first *i *steps *S_i _*(those with absolute value in the interval [*e, e *+ *i*]). Now consider the linearization of the circular permutation such that *e *is the leftmost positive element. To check for each template at step *i *(*f *= *e *+ *i *in this case) we maintain the indices of the following elements visited so far:

• Rightmost positive element: *rp *= max ({|*π*^-1^(| *j*|)| | *j *∈ *S_i_*, *j *> 0})

• Leftmost positive element: *lp *= min ({|*π*^-1^(| *j*|)| | *j *∈ *S_i_*, *j *> 0})

• Rightmost negative element: *rn *= max ({|*π*^-1^(| *j*|)| | *j *∈ *S_i_*, *j *> 0})

• Leftmost negative element: *ln *= min ({|*π*^-1^(| *j*|)| | *j *∈ *S_i_*, *j *> 0})

Template 1 (*eAX *-*f *-*B*) exists, with unsafe reversal *ρ*(*rp *+ 1, *rn*), if and only if the following conditions hold:

1. *lp *= *π*^-1^(|*e*|)

(*e *is the leftmost element visited)

2. *ln *>*rp*

(the negative elements are to the right of the positive)

3. *rn *- *ln *+ *rp *- *lp *= *i *- 1

(the positive and negative elements are all contiguous)

4. *π*^-1^(|*e *+ *i*|) = *ln*

(the last element visited is the leftmost negative element)

5. *i *≥ 3

(the FCI has at least 4 elements)

To check for template 2 we maintain another value *neg *= |{ *j *| *j *∈ *S_i_, j *< 0}|, the number of negative values visited. We know that we have found template 2 (*eA *-*BCf*) with unsafe reversal *ρ*(*ln, rn*) if and only if all of the following conditions hold:

1. *lp *= *π*^-1^(|*e*|)

(*e *is the leftmost element visited)

2. *ln *>*lp*

(the negative elements are to the right of some positive)

3. *rp *>*rn*

(the negative elements are to the left of some positive)

4. *rp *- *lp = i*

(we have visited a contiguous substring)

5. *rn *- *ln = neg *- 1

(the negative elements of *B *are contiguous)

6. *π*^-1^(|*e *+ *i*|) = *rp*

(the last element visited is the rightmost element visited)

7. *i *≥ 3

(the FCI has at least 4 elements)

Note that if at some iteration *i *during our scan conditions 1 or 2 for any of the templates are broken, we know that *e *can no longer match that template.

## 5 The Algorithm

We begin by proving the following theorem.

**Theorem 1 ***For a permutation without a bad component, there is an O*(*n*^2^) *algorithm for listing all sorting reversals*.

**Proof **Use the methods of Section 4 to obtain a blacklist of all ominous substrings associated with each possible smallest frame element *e*. Since the list of all ominous substrings associated with a single smallest frame element is obtained by a linear scan for all possible right endpoints *f*, the time to build the blacklist is *O*(*n*^2^). Each element of the list is associated with a bad reversal, the indices of which we mark in an *n *by *n *matrix; an entry *r *at row *i *and column *j *indicates that the bad reversal *r *acts on elements from position *i *to position *j *in the permutation. Obtain the list of all cycle-splitting reversals in *O*(*n*^2^) time using the standard methods [1]. Finally, examine this list one reversal at a time, removing from the list any reversal that has a corresponding entry marked in the matrix.

The methods described so far are applicable to permutations with no bad components. Permutations with bad components can be easily handled by combining our algorithm with that of Siepel [10] in the following way. First make a linear scan of the permutation to detect bad components [16,17]. If there are bad components, use the *O*(*n*^3^) algorithm of Siepel, otherwise, use our algorithm.

**Theorem 2 ***Pick a signed permutation uniformly at random, the expected time the above algorithm takes to list all sorting reversals is O*(*n*^2^).

**Proof **The probability of seeing a bad component in a permutation taken uniformly at random from the set of all signed permutations is *O*(*n*^-2^) [15]. The bound follows since *n*^3 ^× *n*^-2 ^<*n*^2^.

## 6 Empirical Results

We implemented our ominous substring algorithm in Java (code available from the authors upon request). Preliminary experiments were done comparing the performance of the Java implementation of Siepel's *O*(*n*^3^) algorithm from the package baobabLuna [19] to our average-case *O*(*n*^2^) algorithm. All tests were performed using a 2.16 GHz intel core 2 Duo processor with 1 GB of 667 MHz DDR2 SDRAM.

**Figure 2:**
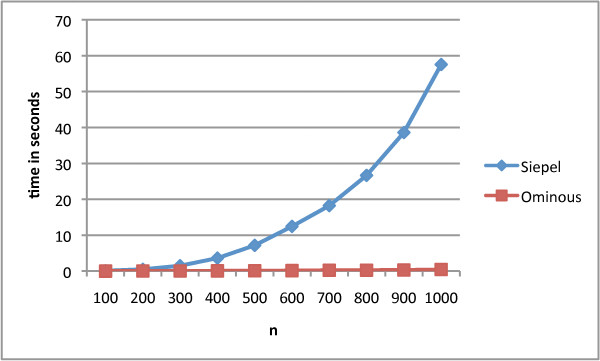
**Running times**. Average running times on random permutations of Siepel and the ominous substrings algorithms for 100 ≤ *n *≤ 1000.

We generated permutations, chosen uniformly at random from the set of all signed permutations, with lengths ranging from *n *= 100 to *n *= 1000. For each value of *n*, 100 experiments were conducted and the average time was reported. Figure 2 shows the savings obtained by applying our new algorithm.

## 7 Conclusions

We presented the first quadratic time algorithm for listing all sorting reversals for a signed permutation. This pattern matching algorithm is simple in that it requires no special data structures. It is optimal in the sense that most permutations have Ω(*n*^2^) sorting reversals [20,21] and since there exists the following family of permutations that have Ω(*n*^2^) unsafe reversals. Take a permutation of length *n *= 2*m *(for any *m*) which is comprised of all the odd numbers positively oriented and in increasing order, followed by all the even numbers in decreasing order but negatively oriented:

There are *m *- 2 reversals that are unsafe where 1 is the left endpoint of a bad component that is created, there are *m *- 3 reversals that are unsafe where 3 is the left endpoint of a bad component that is created, and so on. So there are  unsafe reversals for the permutation of length *n*. This does not discount the possibility of an algorithm that runs in *O*(*n *+ *k*) time where *k *is the number of sorting reversals, although it is currently unclear how to modify our algorithm to obtain this bound.

## 8 Competing interests

The authors declare that they have no competing interests.
